# Adherence to the EAT-Lancet Diet and Its Association with Depression and Anxiety: A Systematic Review and Meta-Analysis of Observational Studies

**DOI:** 10.3390/nu18040684

**Published:** 2026-02-20

**Authors:** Ke-Hong Fang, Ye Lv, Xu-Hui Zhang, Hui Liu, Bing-Zhong Zhai, Yuan Yao, Tian Hu, Hong Xu

**Affiliations:** 1 Department of Health Hazards Surveillance, Hangzhou Center for Disease Control and Prevention (Hangzhou Health Supervision Institution), Hangzhou 310021, China; 2 School of Public Health, Hangzhou Normal University, Hangzhou 311121, China; 15325733635@163.com

**Keywords:** EAT-Lancet diet, depression, anxiety, meta-analysis

## Abstract

Background/Objectives: High-quality diets are increasingly recognized as a promising strategy for alleviating mental health problems. This study aimed to evaluate the association between adherence to the EAT-Lancet diet (ELD) and depression and anxiety using a systematic review and meta-analysis of observational studies. Methods: A comprehensive literature search of PubMed, Web of Science, and Embase was conducted. Two independent reviewers screened titles, abstracts, and full texts and extracted data, with any discrepancies resolved by consensus. Results: Ultimately, eight unique studies (10 comparisons) met the predefined inclusion criteria. Adjusted effect estimates (odds ratios (ORs) or hazard ratios (HRs)) and corresponding 95% confidence (95%CI) intervals were extracted and pooled using random-effects meta-analysis. Between-study heterogeneity was quantified with the *I*^2^ statistic. Compared with the lowest ELD adherence, the highest adherence was associated with a lower risk of depression (OR = 0.78, 95% CI 0.73–0.85; *I*^2^ = 85.0%); a similar inverse association was observed for anxiety (OR = 0.83, 95% CI 0.79–0.86; *I*^2^ = 0%) and the co-occurrence of depression and anxiety (HR = 0.76, 95% CI 0.68–0.85; *I*^2^ = 0%). Conclusions: Our findings indicate that, compared with low adherence, high adherence to the ELD is associated with beneficial effects on mental health and may inform dietary recommendations for the early prevention and intervention of depression, anxiety, and their co-occurrence.

## 1. Introduction

The World Health Organization defines health as more than the absence of physical disease, encompassing mental well-being, moral wellbeing, and satisfactory social functioning [1], thereby underscoring the importance of mental health. Epidemiological data indicate that approximately one third of individuals will experience at least one mental disorder during their lifetimes [2]. Global Burden of Disease estimates show that the disability-adjusted life years (DALYs) attributable to mental disorders rose substantially from 1990 to 2019 (moving from the 13th to the 7th leading cause) [3], and, in 2021, roughly 155 million DALYs were attributable to mental health conditions [4]. Among mental health disorders, depression and anxiety impose the greatest burden, accounting for 28.9% and 31.0% of the total, respectively [5].

Given the substantial health burden attributable to depression and anxiety, identifying modifiable risk factors for primary prevention is of major public health importance. Recent evidence indicates that nutrients can affect mental health by modulating neuroendocrine function [6,7]. Compared with studies of individual nutrients or single foods, dietary pattern analyses more effectively capture the complex combinations and potential synergistic effects of whole diets and therefore offer greater explanatory power when assessing the relationships between diet and mental health outcomes such as depression and anxiety [8,9,10].

Against the dual backdrop of substantial environmental harm from global food production [11,12] and the reciprocal threat that environmental degradation poses to human health [13], in 2019, the EAT-Lancet Commission proposed the EAT-Lancet diet (ELD) [11]. The ELD advocates for a predominantly plant-based dietary pattern, emphasizing increased consumption of vegetables, fruits, whole grains, legumes, and nuts; moderate inclusion of seafood and poultry; and stringent limitations on red meat, added sugars, and saturated fats. It aims to meet nutritional requirements within the planet’s ecological carrying capacity [11].

Although environmentally sustainable and health-promoting diets (e.g., ELD) that balance human health with planetary sustainability have received increased attention, adherence to the ELD in particular has been associated with reduced risks of certain chronic diseases [14,15]. However, to our knowledge, empirical studies examining the relationship between ELD adherence and depression, anxiety, and their comorbidity remain limited. In light of the growing global burden of mental disorders and the potential for dietary interventions to prevent and alleviate psychological conditions, the systematic synthesis and quantitative appraisal of the current evidence is warranted.

In this study, we undertake a systematic review and meta-analysis to comprehensively summarize and evaluate the association between adherence to the ELD and the risks of depression, anxiety, and their comorbidity. We aim to fill existing gaps in the literature and to provide an evidence base to inform clinical practice and public health interventions.

## 2. Materials and Methods

The reporting of this meta-analysis follows the Meta-Analysis of Observational Studies in Epidemiology (MOOSE) statement [16] (Table S1) and the Preferred Reporting Items for Systematic Reviews and Meta-Analyses (PRISMA) guidelines [17] (Table S2). The study protocol was registered and approved on the International Prospective Register of Systematic Reviews (PROSPERO) (registration number: CRD420251251327).

### 2.1. Search Strategy

A systematic and comprehensive literature search was conducted across three electronic databases—PubMed, Web of Science, and Embase—up to 12 December 2025. The search codes are described in detail in Table S3. Furthermore, the reference lists of all eligible studies were manually reviewed. We did not search the grey literature or contact authors for Supplementary Information, as the required statistics were sufficiently provided in the primary publications. Only studies published in English were considered for inclusion.

### 2.2. Inclusion Criteria and Exclusion Criteria

Inclusion criteria were defined as follows: (a) an original study examining the relationship between the ELD and depression/anxiety; (b) a longitudinal/prospective cohort study, case–control study, or cross-sectional study design; (c) odds ratios (ORs), relative risks (RRs), or hazard ratios (HRs) with at least a 95% confidence interval (95% CI) (or the data with which to calculate them) were present; (d) the definitions of the ELD and depression/anxiety were declared. Exclusion criteria were the following: (a) insufficient data on the ELD and depression/anxiety; (b) the study was a review or commentary article. To ensure the independence of our analytical units, we excluded duplicate publications and studies with significant overlap in original data. Two researchers (K.-H.F. and X.-H.Z.) independently performed study selection by screening the titles, abstracts, and subsequently the full texts of the identified records. Any differences in selection were resolved by consensus or through discussion with a third investigator (Y.L.).

### 2.3. Data Extraction

Two researchers (K.-H.F. and B.-Z.Z.) independently designed Microsoft Excel spreadsheets to extract the study data and subsequently cross-checked the results. All inconsistencies were resolved through discussion between the two researchers; if no consensus could be reached, the final decision was arbitrated by a third researcher (H.L.). The extracted data encompassed (a) the first author’s name, publication year, and country of origin; (b) the study design; (c) participant characteristics (sample size and age); (d) assessment tools for the ELD and depression/anxiety; (e) exclusion criteria; and (f) covariates adjusted for in the multivariate analyses.

For studies that reported multiple outcomes based on different ELD scoring systems, each scoring method was treated as an independent dataset in the meta-analysis to maximize the capture of the available evidence. Specifically, for the study by Lu et al. [18], which employed three different scoring methods to assess ELD adherence, each method was treated as an independent dataset in the meta-analysis to comprehensively evaluate the impacts of different scoring interpretations.

### 2.4. Quality Assessment

Two researchers (T.H. and L.H.) assessed the quality of each selected article using the Newcastle–Ottawa Scale (NOS), which evaluates non-randomized studies in systematic reviews and meta-analyses across three domains: selection, comparability, and outcomes. For cross-sectional studies [19], the maximum score is 10, with 9–10 points classified as very good, 7–8 points as good, 5–6 points as satisfactory, and 0–4 points as unsatisfactory. For cohort studies [20], the maximum score is 9, with 7–9 points indicating high quality, 4–6 points moderate quality, and 0–3 points low quality. Discrepancies were resolved by consensus between the two reviewers; if agreement could not be reached, a third reviewer (H.L.) made the final decision.

### 2.5. Grading of Evidence

The certainty of evidence for each outcome was evaluated using the Grading of Recommendations, Assessment, Development, and Evaluation (GRADE) framework [21], which categorizes the quality of evidence into four levels: high, moderate, low, or very low. Following the standard GRADE methodology, the evidence was assessed across five domains: risk of bias (based on Newcastle–Ottawa Scale scores), inconsistency (downgraded for “serious” (50% ≤ *I*^2^ < 75%) or “very serious” (*I*^2^ ≥ 75%) heterogeneity), indirectness (considering the applicability of evidence to the research question), imprecision (evaluated based on whether the 95% confidence interval was wide, crossed the null effect line, or indicated high uncertainty with an insufficient sample size), and other considerations (including publication bias, dose–response gradients, and effect magnitudes). The assessment was performed independently by two reviewers (K.-H.F. and Y.Y.), with any discrepancies resolved through consensus, and the final evidence profiles were generated using the GRADEpro GDT online tool (https://gdt.gradepro.org/, accessed 9 February 2026).

### 2.6. Data Analysis

For the meta-analysis, pooled odds ratios (ORs) or hazard ratios (HRs) were calculated to compare the associations between the highest and lowest adherence to the ELD and depression, anxiety, or their comorbidity. These analyses were performed separately for unadjusted estimates and for estimates adjusted for potential confounders. Random effects was employed to synthesize the effect sizes. Heterogeneity across studies was estimated using the Paule and Mandel estimator and quantified with the *I*^2^ statistic [22]. To facilitate the clinical interpretation of heterogeneity, prediction intervals (PIs) were also reported to indicate the expected range of effect sizes in future studies [23]. We employed funnel plot asymmetry and Egger’s test to assess potential publication bias [24]; however, given that the statistical power of these tests is often limited when the number of included studies is small, with a commonly recommended threshold of at least 10 studies, findings for outcomes with fewer than this threshold were regarded as exploratory. Furthermore, subgroup analyses and sensitivity analyses were conducted to explore potential sources of heterogeneity and to evaluate the robustness of the pooled results, respectively. All statistical analyses were performed using the R software (version 4.4.1), with a *p*-value < 0.05 considered statistically significant.

## 3. Results

### 3.1. Literature Search, Study Characteristics, and Quality Assessment

**Figure 1:**
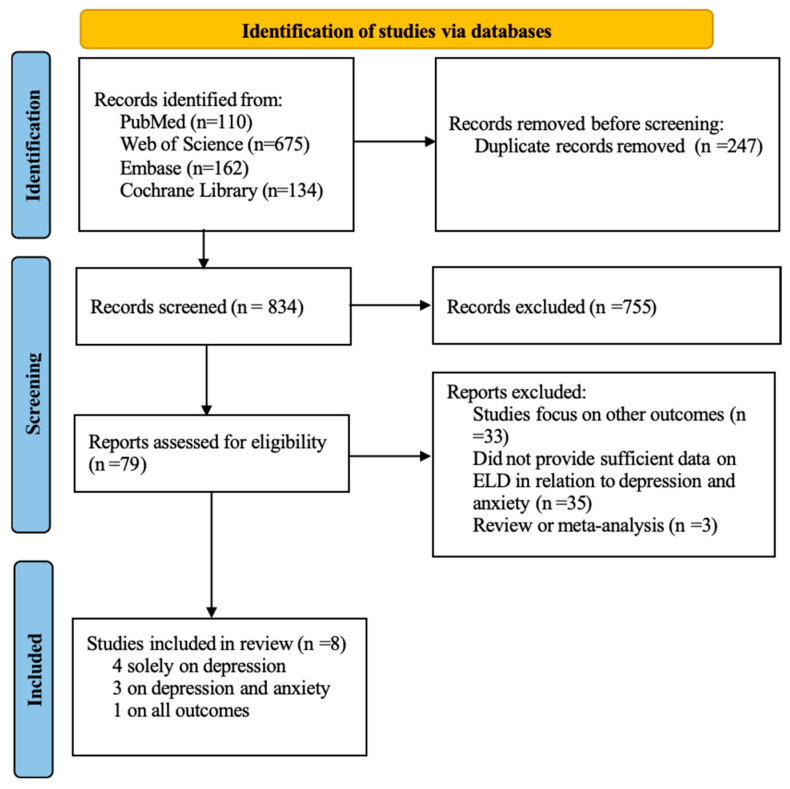
Flowchart of study screening and selection process.

A systematic search of the PubMed, Web of Science, and Embase databases yielded 1081 records. After the removal of 247 duplicates using Zotero 7, 834 articles remained for title and abstract screening, during which 755 studies were excluded. Subsequently, 79 full-text articles were assessed for eligibility. Finally, eight unique studies meeting the inclusion criteria were included in the analysis [18,25,26,27,28,29,30,31]: four solely on depression, three on both depression and anxiety, and one on both depression, anxiety, and their co-occurrence. The detailed literature selection process is illustrated in the flow diagram (Figure 1).

A critical refinement was included to ensure the granularity of the synthesis: since Lu et al. [18] reported outcomes based on three distinct ELD scoring systems, these were treated as three independent datasets in our analysis. Consequently, while the meta-analysis comprises eight unique studies, it incorporates a total of 10 independent comparisons for the quantitative synthesis. To maintain clarity in the forest plots, these partitions are distinguished by alphabetical suffixes [18].

Among the eight included studies, five utilized a cross-sectional design and three were prospective cohort studies, encompassing a total pooled population exceeding 400,000 participants. Regarding outcome distribution, eight studies (10 comparisons) reported on depression, four studies (six comparisons) on anxiety, and one study (three comparisons) on the co-occurrence of depression and anxiety. The primary characteristics of these studies are detailed in Table 1.

The methodological quality of the included studies, as assessed by the Newcastle–Ottawa Scale (NOS), is summarized in Tables S4 and S5. For the cross-sectional studies, the scores ranged from 6 to 8, indicating a quality level ranging from “satisfactory” to “good” (Table S4). Among the cohort studies, all quality scores were 8, with all studies being categorized as “high quality” (Table S5). These studies performed well in the assessment of outcomes, and no study was excluded from the meta-analysis due to poor methodological quality.

### 3.2. Outcomes of Interest

#### 3.2.1. ELD Adherence and Depression

**Figure 2:**
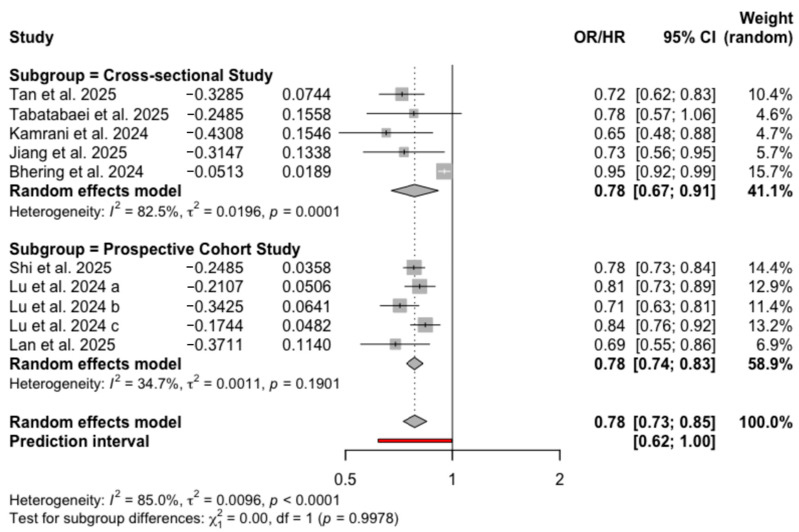
Forest plot of adjusted associations of ELD and depression [18,25,26,27,29,30,31,32].

Before adjustment for confounders, six unique studies—yielding eight independent comparisons (as Lu et al. [18] provided three)—were included; two studies [25,26] were excluded from this specific analysis as they did not report unadjusted data. This meta-analysis of eight comparisons indicated that the highest ELD adherence was associated with a significant reduction in depression risk (OR = 0.70, 95% CI: 0.60–0.82, *I*^2^ = 87.5%, PI: 0.41–1.19), as shown in Figure S1. After adjustment for confounding factors, the synthesis incorporated all eight unique studies, providing a total of 10 independent comparisons. The fully adjusted model demonstrated that the highest level of ELD adherence was associated with a lower risk of depression compared to the lowest adherence (OR = 0.78, 95% CI: 0.73–0.85, *I*^2^ = 85.0%, PI: 0.62–1.00), with the results presented in Figure 2. When stratified by study design, the pooled effect sizes were OR = 0.78 (95% CI: 0.67–0.91, *I*^2^ = 82.5%) for cross-sectional studies and HR = 0.78 (95% CI: 0.74–0.83, *I*^2^ = 34.7%) for prospective cohort studies (Figure 2). Gender-stratified subgroup analyses yielded comparable inverse associations between ELD adherence and depression for men (OR = 0.88, 95% CI: 0.86–0.91, *I*^2^ = 0.0%) and women (OR = 0.86, 95% CI: 0.79–0.93, *I*^2^ = 58.6%), although heterogeneity was notably higher in the female subgroup (Figure S2). Finally, according to the GRADE framework, the overall certainty of evidence for the association between ELD adherence and depression was rated as moderate. The quality of evidence was downgraded due to substantial heterogeneity and potential publication bias (Table S6).

#### 3.2.2. ELD Adherence and Anxiety

**Figure 3:**
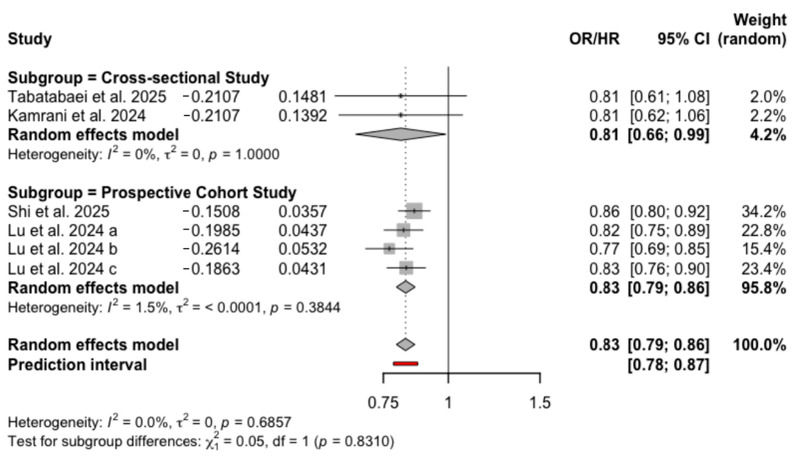
Forest plot of adjusted associations of ELD and anxiety [18,26,30,32].

Prior to adjustment for confounders, three unique studies provided five independent comparisons (Lu et al. [18] contributed three). One study [26] was excluded from this analysis because unadjusted estimates were not reported. The crude analysis showed that higher ELD adherence was inversely associated with anxiety (OR = 0.91, 95% CI: 0.87–0.96, *I*^2^ =49.5%, PI: 0.75–1.12) (Figure S3). After adjustment for confounding factors, all four unique studies were included, yielding six independent comparisons. The fully adjusted model indicated that individuals with the highest ELD adherence had a significantly lower likelihood of anxiety compared to those with low adherence (OR = 0.83, 95%CI: 0.79–0.86, *I*^2^ = 0.0%, PI: 0.78–0.97), as illustrated in Figure 3. In the subgroup analysis by study design, the pooled estimates were OR = 0.81 (95% CI: 0.66–0.99, *I*^2^ = 0%) for cross-sectional studies and HR = 0.83 (95% CI: 0.79–0.86, *I*^2^ = 1.5%) for prospective cohort studies (Figure 3). Gender-stratified analyses showed that ELD adherence was associated with lower odds of anxiety in men (OR = 0.88, 95% CI: 0.80–0.96, *I*^2^ = 0.0%), but not in women (OR = 0.86, 95% CI: 0.68–1.10, *I*^2^ = 77.0%) (Figure S4). Finally, according to the GRADE assessment, the overall certainty of evidence for the association between the ELD and anxiety was rated as moderate (Table S6). 

#### 3.2.3. ELD Adherence and the Co-Occurrence of Depression and Anxiety

**Figure 4:**
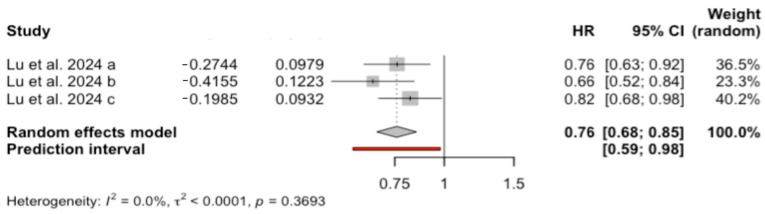
Forest plot of adjusted associations of ELD and depression–anxiety co-occurrence [18].

For the analysis of depression–anxiety comorbidity, one unique study [18] provided three independent comparisons for synthesis. Under unadjusted confounding factors, ELD adherence showed a significant negative association with the risk of depression–anxiety comorbidity (HR = 0.77, 95% CI: 0.65–0.91, *I*^2^ = 54.5%, PI: 0.42–1.40) (Figure S5). After adjustment for confounding factors, the meta-analysis results indicated that, compared with the population with lower ELD adherence, those with the highest adherence had a significantly reduced risk of depression–anxiety comorbidity (HR = 0.76, 95% CI: 0.68–0.85, *I*^2^ =0.0%, PI: 0.59–0.98) (Figure 4). Gender-stratified analyses showed that adherence to the ELD was negatively associated with the co-occurrence of depression and anxiety in women (HR = 0.72, 95% CI: 0.62–0.83, *I*^2^ = 17.4%), but not in men (HR = 0.92, 95% CI: 0.76–1.12, *I*^2^ = 0.0%) (Figure S6). According to the GRADE framework, the overall certainty of evidence for the association between ELD adherence and the co-occurrence of depression and anxiety was rated as very low. The evidence was downgraded primarily due to the inclusion of non-randomized studies and imprecision (Table S6).

### 3.3. Publication Bias Assessment and Sensitivity Analysis

In the assessment of publication bias regarding the association between ELD adherence and depression, the adjusted funnel plot revealed pronounced asymmetry, with data points predominantly clustered on the left side (Figure S7). Egger’s test confirmed the presence of significant publication bias (*p* = 0.0050). In contrast, the funnel plots for ELD adherence in relation to anxiety and depression–anxiety comorbidity displayed symmetric distributions, and Egger’s tests indicated no evidence of publication bias, with all *p*-values > 0.05.

The sensitivity analysis results demonstrated that, when evaluating the robustness of the association between ELD adherence and depression, individual studies exerted a substantial influence on result heterogeneity. Specifically, using the leave-one-out method to sequentially exclude each study, omitting the study by Bhering et al. [25] led to a decrease in the *I*^2^ from 85.0% to 10.8%. However, in the sensitivity analysis assessing the association between the ELD and anxiety, no comparable heterogeneity variations were identified.

### 3.4. Subgroup Analyses

Subgroup analyses were conducted based on the sample size, country, and depression/anxiety assessment tools (Table 2). For depression, with stratification by sample size, a significant inverse association was observed only in studies with ≥10,000 participants (OR = 0.78, 95% CI: 0.75–0.81; *I*^2^ = 21.3%, *p* = 0.267). By country, significant associations were found in studies from the United States (OR = 0.71, 95% CI: 0.64–0.80; *I*^2^ = 0.0%, *p* = 0.937) and the United Kingdom (OR = 0.79, 95% CI: 0.75–0.83; *I*^2^ = 37.1%, *p* = 0.190). With respect to depression assessment tools, the association appeared stronger when using the PHQ-9 (OR = 0.78, 95% CI: 0.75–0.81; *I*^2^ = 21.3%, *p* = 0.267). For anxiety, stronger inverse associations with ELD adherence were observed in studies with ≥10,000 participants, those conducted in the United Kingdom, and those using the GAD-7 for assessment (OR = 0.83, 95% CI: 0.79–0.86; *I*^2^ = 1.5%, *p* = 0.384).

## 4. Discussion

To our knowledge, this study is the first systematic review and meta-analysis to specifically evaluate the association between the ELD and depression, anxiety, and their comorbidity. We included eight relevant studies and systematically synthesized and pooled the available adjusted effect estimates. Overall, the findings support an inverse association between adherence to the ELD and depression, anxiety, and their co-occurrence. Subgroup analyses of prospective cohort studies indicated that, compared with those with lower adherence, participants with higher adherence to the ELD had an approximately 22% lower risk of depression and a 17% lower risk of anxiety.

Our findings are consistent with previous reviews indicating that adherence to high-quality dietary patterns, such as the Healthy Eating Index (HEI), the Alternative Healthy Eating Index (AHEI), the Mediterranean diet, and the DASH diet, is associated with lower risks of depression and anxiety [33,34,35,36,37,38]. Dietary patterns that emphasize plant-based foods while allowing moderate amounts of certain animal products appear particularly beneficial. Both the HEI and AHEI stress the consumption of fruits, vegetables, whole grains, and unsaturated fats, which are also core components of the ELD [39,40]. Furthermore, high adherence to a “healthy plant-based” dietary pattern, which is characterized by an emphasis on healthy plant foods and by restricting unhealthy plant foods and some animal foods, has been significantly associated with lower anxiety, depression, and psychological distress [41].

Results from prospective cohort studies provide stronger evidence for an inverse association between adherence to the ELD and lower risks of depression and anxiety. Compared with cross-sectional studies, prospective designs can partly mitigate concerns about reverse causation (i.e., depression/anxiety leading to poor dietary choices), rendering these findings more convincing from a causal perspective. Nevertheless, residual confounding may remain even after multivariable adjustment. Moreover, prospective cohorts varied in terms of exposure assessment (ELD scoring) and outcome measurement (clinical diagnoses versus screening instruments), follow-up duration, and the selection of covariates. Although subgroup heterogeneity was relatively low in our analyses, further high-quality cohort studies or randomized dietary intervention trials that use a standardized ELD assessment, have longer follow-up periods, and rigorously control for potential confounders are needed to strengthen causal inferences and to clarify the underlying biological mechanisms.

Gender-stratified analyses revealed an intriguing differential pattern: the inverse association between ELD adherence and anxiety was mainly observed in men, whereas the association with depression–anxiety comorbidity was more pronounced in women. These gender differences may reflect a combination of biological, psychological, and social factors [23,38,39,40,41,42,43,44]. For example, men and women differ in the levels of hormones that modulate inflammatory responses and the gut microbiome composition (e.g., estrogen, testosterone) [45]. In addition, gender-related differences in dietary adherence, stress-coping strategies, and social determinants of health may have contributed to the heterogeneous associations observed [30,46]. However, because relatively few studies reported gender-stratified data, these findings should be interpreted with caution and warrant further research to confirm and elucidate the underlying mechanisms.

Sensitivity analyses indicated that the study by Bhering et al. [25] was a major source of heterogeneity in the depression analysis: excluding this study reduced the *I*^2^ from 85.0% to 10.8%. Several factors may account for this discrepancy. Bhering et al.’s work was reported only as a conference abstract, with methodological details and results not fully described or peer-reviewed, and they employed a diagnostic interview (the Diagnostic Interview for Genetic Studies), whereas most other studies used symptom screening instruments (e.g., PHQ-9, GAD-7). Diagnostic interviews and screening scales differ fundamentally in construct, rigor, and outcome classification, which can produce systematic differences in effect estimates.

The biological mechanisms underpinning the association remain incompletely understood but likely involve multiple interrelated physiological pathways. First, systemic inflammation and oxidative stress are implicated in the pathogenesis of depression and anxiety; elevated inflammatory markers can impair neuroplasticity and disrupt the functioning of neurotrophic factors [29]. Predominantly plant-based diets are associated with reductions in systemic inflammation [46,47,48,49], whereas high-energy, high-saturated-fat diets promote free-radical formation and oxidative stress and compromise hippocampal integrity and the blood–brain barrier; thus, they may adversely affect mood and cognitive regulation [30,46,50,51,52]. Second, adherence to the ELD tends to increase the intake of dietary fiber, potassium, selenium, and other micronutrients [29,53,54]; these nutrients are essential for neurotransmitter metabolism and receptor expression and have been linked to improvements in behavior, cognition, memory, and affect when adequately supplied [55,56]. Third, the gut–brain axis provides a key mechanistic route: components of the ELD, such as fiber and polyphenols, can reshape the gut microbiota and promote the production of microbial metabolites such as short-chain fatty acids that exert anti-inflammatory and neuroregulatory effects via immune, neural, and endocrine pathways, thereby influencing central nervous system function, mood, and behavior [57,58,59,60,61].

The principal strengths of this study are as follows. To our knowledge, this is the first systematic meta-analysis examining the associations of the ELD with depression, anxiety, and their comorbidity, and it includes a pooled sample of over 400,000 participants, which substantially increases the statistical power and the generalizability of the findings. Most included studies were rated as high-quality, bolstering the credibility of the evidence. Methodologically, we synthesized multivariable-adjusted effect estimates from individual studies to account for known confounders and conducted sensitivity analyses to verify the robustness of the primary results. The findings have clear clinical and public health implications: they provide evidence in support of dietary approaches for the primary prevention of depression and anxiety and for individualized nutritional interventions, and they may inform the incorporation of ELD principles into clinical practice and population-level prevention strategies. Finally, we performed multiple subgroup analyses (by study design, country/region, gender, sample size, and outcome assessment method) to explore sources of heterogeneity and to identify potentially susceptible subgroups, thereby offering guidance for future targeted intervention studies and policy development.

This study has several limitations. First, the limited availability of data precluded dose–response analyses, preventing the quantitative characterization of the relationship between adherence to the ELD and the risks of depression, anxiety, and their comorbidity. Second, the included studies were heterogeneous with respect to geographic location and population composition and were predominantly conducted in European prospective cohorts, which may limit the global generalizability of the findings. Third, evidence of publication bias was detected in the depression analyses, raising the possibility that the observed association may be overestimated. Finally, the evidence base for anxiety and for depression–anxiety comorbidity was sparse (only four and one studies, respectively), and gender-stratified data were limited, reducing the robustness and external validity of these particular results. These limitations indicate that future research should broaden the geographic and demographic coverage, perform rigorous dose–response assessments, and incorporate more comprehensive gender-specific analyses to validate and refine the present findings.

## 5. Conclusions

This systematic review and meta-analysis of observational studies indicates that higher adherence to the ELD is inversely associated with risks of depression, anxiety, and their comorbidity. The findings suggest that adopting a planetary health dietary pattern that is predominantly plant-based, while limiting red meat and added sugars, may confer protective effects for population mental health. Analyses stratified by gender revealed gender-specific differences in the associations between diet adherence and mental health outcomes, providing important insights for precision nutrition interventions and mechanistic research. Future studies should prioritize high-quality prospective cohort studies, randomized controlled trials, and cross-cultural comparisons; harmonize dietary assessment methods; and further elucidate the underlying biological and behavioral mechanisms to establish a robust evidence base for public health strategies that promote both mental health and planetary sustainability.

## Figures and Tables

**Table 1:** Characteristics of the included studies.

Author and Year	Country	Study Design/Source of Data	Population	Female (%)	Age	ELD Assessment Tool	Depression Assessment Tool	Anxiety Assessment Tool	Exclusion Criteria	Adjusted Variables
Tan et al. 2025 [31]	United States	Cross-sectional study	27,756	12,545 (45.20)	≥20	PHDI-US	PHQ-9	NA	(1) Age below 20 years; (2) missing depression assessment data; (3) missing dietary data; and (4) missing key covariates	Age, gender, race, PIR, education, BMI, smoking, and hypertension
Tabatabaei et al. 2025 [30]	Iran	Cross-sectional study	1994	1043 (52.31)	≥18	ELD	HADS	HADS	(1) With comorbid systemic or metabolic diseases; (2) with abnormal energy intake; and (3) adhering to any special diets	Age, sex, energy, marital status, smoking, physical activity, socioeconomic status, and center effect
Shi et al. 2025 [26]	UK	Prospective cohort study/UK Biobank cohort	169,916	91,619 (53.90)	≥40	PHDI	PHQ-9	GAD-7	(1) Missing dietary data; (2) baseline diagnosis of neurological or psychiatric disorder; and (3) unreliable questionnaire data	Age, sex, ethnicity, smoking status, alcohol consumption status (not for alcohol), physical activity, TDI, total energy intake, education level, assessment center, healthy sleep duration, whether living alone, and family history of disease
Lu et al. 2024 [18]	UK	Prospective cohort study/UK Biobank cohort	180,446	96,622 (53.55)	37–73	Knuppel ELD, Stubbendorff ELD, Kesse-Guyot ELD	PHQ-9	GAD-7	(1) Withdrew from the survey; (2) suffered from depression or anxiety at baseline; (3) reported use of anxiolytics or antidepressants at baseline; and (4) with abnormal total energy intakes	Age, sex, Townsend scores, ethnicity, smoking status, alcohol intake, physical activity, hypertension, BMI, and total energy intake
Lan et al. 2025 [27]	United States	Prospective cohort study/NHANES	25,312	13,182 (52.08)	≥20	PHDI	PHQ-9	NA	(1) Missing depression data; (2) missing PHDI data; (3) missing follow-up time data; and (4) those with incomplete covariate data	Age, sex, race/ethnicity, marital status, education, PIR, smoking, drinking, physical activity, BMI, and uric acid
Kamrani et al. 2024 [32]	Iran	Cross-sectional study	4579	3520 (77.00)	30–70	PHDI	DASS-21	DASS-21	Missing data for MIND diet calculation	Age, sex, BMI, physical activity, education level, smoking, alcohol, drugs, residence type, ethnicity, family history of psychiatric disorders, and energy intake
Jiang et al. 2025 [29]	United States	Cross-sectional study	30,446	15,373 (50.49)	≥20	PHDI	PHQ-9	NA	(1) Under the age of 20, pregnant women; (2) knowledge deficits related to depression; and (3) missing PHDI data	Age, gender, education level, marital status, PIR, race, obesity, smoking, activity, hypertension, diabetes, and high cholesterol
Bhering et al. 2024 [25]	Switzerland	Cross-sectional study	3473	NA	NA	ELD	Diagnostic Interview for Genetic Studies	NA	NA	NA

Abbreviations: ELD: EAT-Lancet diet; PHDI: Planetary Health Diet Index; US: the United States; PHQ-9: Patient Health Questionnaire 9; NA: Not Available; PIR: poverty-to-income ratio; BMI: body mass index; HADS: Hospital Anxiety and Depression Scale; UK: the United Kingdom; GAD-7: Generalized Anxiety Disorder—7 items; TDI: Townsend Deprivation Index; NHANES: National Health and Nutrition Examination Survey; DASS-21: Depression, Anxiety, and Stress Scale—21 items; MIND: Mediterranean-DASH Intervention for Neurodegenerative Delay.

**Table 2:** Subgroup analyses of ELD and depression and anxiety.

Subgroup	Depression				Anxiety			
	OR (95%CI)	*I*^2^ Value (%)	*p* Value	N	OR (95%CI)	*I*^2^ Value (%)	*p* Value	N
Sample size								
≥10,000	0.78 (0.75–0.81)	21.3%	0.267	7	0.83 (0.79–0.86)	1.5%	0.384	4
<10,000	0.82 (0.65–1.03)	73.1%	0.024	3	0.81 (0.66–0.99)	0.0%	1.000	2
Country								
United States	0.71 (0.64–0.80)	0.0%	0.937	3	NA	NA	NA	NA
UK	0.79 (0.75–0.83)	37.1%	0.190	4	0.83 (0.79–0.86)	1.5%	0.384	4
Others	0.82 (0.65–1.03)	73.1%	0.024	3	0.81 (0.66–0.99)	0.0%	1.000	2
Depression Assessment Tool								
PHQ-9	0.78 (0.75–0.81)	21.3%	0.267	7	NA	NA	NA	NA
Others	0.82 (0.65–1.03)	73.1%	0.024	3	NA	NA	NA	NA
Anxiety Assessment Tool								
GAD-7	NA	NA	NA	NA	0.83 (0.79–0.86)	1.5%	0.384	4
Others	NA	NA	NA	NA	0.81 (0.66–0.99)	0.0%	1.000	2

NA: Not Available.

## Data Availability

This study made use of publicly available data from published studies; therefore, no original data are available for sharing.

## Supplementary Materials

The following supporting information can be downloaded at https://www.mdpi.com/article/10.3390/nu18040684/s1. Table S1: MOOSE Checklist; Table S2: PRISMA Checklist; Table S3: Search strategy; Table S4: Quality assessment using the Newcastle-Ottawa scale for cross-sectional studies; Table S5: Quality assessment using the Newcastle-Ottawa scale for cohort studies; Table S6: GRADEPro assessment of the certainty of evidence regarding ELD adherence and its association with depression and anxiety; Figure S1: Forest plot of unadjusted associations of ELD and depression; Figure S2: Forest plot of adjusted associations of ELD and depression by gender; Figure S3: Forest plot of unadjusted associations of ELD and anxiety; Figure S4: Forest plot of adjusted associations of ELD and anxiety by gender; Figure S5: Forest plot of unadjusted associations of ELD and co-occurrence; Figure S6: Forest plot of adjusted associations of ELD and co-occurrence by gender; Figure S7: Funnel plots for assessing publication bias in studies on ELD and depression/anxiety.
