# Supplementary material for: Adherence to the EAT-Lancet Diet and Its Association with Depression and Anxiety: A Systematic Review and Meta-Analysis of Observational Studies
Source: Nutrients. 2026 Feb 20;18(4):684. doi: 10.3390/nu18040684 (PMC12943308; doi:10.3390/nu18040684)
Supplement: Supplementary file 1 [file nutrients-18-00684-s001.zip › nutrients-4147444-supplementary.pdf]

Supplementary appendix

Table S1. MOOSE Checklist (p2)
Table S2. PRISMA Checklist (p4)
Table S3. Search strategy (p9)
Table S4. Quality assessment using the Newcastle-Ottawa scale for cross-sectional studies (p10)
Table S5. Quality assessment using the Newcastle-Ottawa scale for cohort studies (p10)
Table S6. GRADEPro assessment of the certainty of evidence regarding ELD adherence and its association with depression and anxiety (p11)
Figure S1. Forest plot of unadjusted associations of ELD and depression (p12)
Figure S2. Forest plot of adjusted associations of ELD and depression by gender (p12)
Figure S3. Forest plot of unadjusted associations of ELD and anxiety (p13)
Figure S4. Forest plot of adjusted associations of ELD and anxiety by gender (p13)
Figure S5. Forest plot of unadjusted associations of ELD and co-occurrence (p13)
Figure S6. Forest plot of adjusted associations of ELD and co-occurrence by gender (p14)
Figure S7. Funnel plots for assessing publication bias in studies on ELD and depression/anxiety (p14)

Table S1: MOOSE Checklist

Item No	Recommendation	Reported on Page No
**Reporting of background should include**		
1	Problem definition	1
2	Hypothesis statement	1
3	Description of study outcome(s)	1,3
4	Type of exposure or intervention used	1,3
5	Type of study designs used	1,3,5
6	Study population	1, Table 1
**Reporting of search strategy should include**		
7	Qualifications of searchers (eg, librarians and investigators)	3-4
8	Search strategy, including time period included in the synthesis and keywords	3-4, Supplemental Table S3
9	Effort to include all available studies, including contact with authors	3
10	Databases and registries searched	3
11	Search software used, name and version, including special features used (eg, explosion)	3, Supplemental Table S3
12	Use of hand searching (eg, reference lists of obtained articles)	3
13	List of citations located and those excluded, including justification	3, Figure 1
14	Method of addressing articles published in languages other than English	3
15	Method of handling abstracts and unpublished studies	3
16	Description of any contact with authors	3
**Reporting of methods should include**		
17	Description of relevance or appropriateness of studies assembled for assessing the hypothesis to be tested	3,Table 1, Supplemental Table S3
18	Rationale for the selection and coding of data (eg, sound clinical principles or convenience)	3-4, Table 1

19	Documentation of how data were classified and coded (eg, multiple raters, blinding, and interrater reliability)	3-4
20	Assessment of confounding (eg, comparability of cases and controls in studies where appropriate)	4, Table 1
21	Assessment of study quality, including blinding of quality assessors; stratification or regression on possible predictors of study results	4, Supplemental Table S4-S6
22	Assessment of heterogeneity	4-5
23	Description of statistical methods (eg, complete description of fixed or random effects models, justification of whether the chosen models account for predictors of study results, dose-response models, or cumulative meta-analysis) in sufficient detail to be replicated	1, 4-5
24	Provision of appropriate tables and graphics	Figure 1-4, Table 1-2, Supplemental Figure S1-S7
**Reporting of results should include**		
25	Graphic summarizing individual study estimates and overall estimate	Figure 2-4, Supplemental Figure S1-S6
26	Table giving descriptive information for each study included	Table 1
27	Results of sensitivity testing (eg, subgroup analysis)	10, Table 2
28	Indication of statistical uncertainty of findings	8-10
**Reporting of discussion should include**		
29	Quantitative assessment of bias (eg, publication bias)	12, Supplemental Table 4-6, Supplemental Figure S7
30	Justification for exclusion (eg, exclusion of non–English-language citations)	3
31	Assessment of quality of included studies	11-13
**Reporting of conclusions should include**		
32	Consideration of alternative explanations for observed results	11-13
33	Generalisation of the conclusions (ie, appropriate for the data presented and within the domain of the literature review)	12-13
34	Guidelines for future research	12-13
35	Disclosure of funding source	13

Table S2: PRISMA Checklist

Section and Topic	Item	Checklist item	Location where item is reported (p)
**TITLE**			
Title	1	Identify the report as a systematic review.	1
**ABSTRACT**			
Abstract	2	See the PRISMA 2020 for Abstracts checklist.	1
**INTRODUCTION**			
Rationale	3	Describe the rationale for the review in the context of existing knowledge.	2-3
Objectives	4	Provide an explicit statement of the objective(s) or question(s) the review addresses.	3
**METHODS**			
Eligibility criteria	5	Specify the inclusion and exclusion criteria for the review and how studies were grouped for the syntheses.	4
Information sources	6	Specify all databases, registers, websites, organisations, reference lists and other sources searched or consulted to identify studies. Specify the date when each source was last searched or consulted.	3
Search strategy	7	Present the full search strategies for all databases, registers and websites, including any filters and limits used.	3, Supplemental Table S3
Selection process	8	Specify the methods used to decide whether a study met the inclusion criteria of the review, including how many reviewers screened each record and each report retrieved, whether they worked independently, and if applicable, details of automation tools used in the process.	3-4, Figure 1
Data collection	9	Specify the methods used to collect data from reports, including how many reviewers collected data from each report, whether they worked independently, any	3-4

Section and Topic	Item	Checklist item	Location where item is reported (p)
process		processes for obtaining or confirming data from study investigators, and if applicable, details of automation tools used in the process.	
Data items	10a	List and define all outcomes for which data were sought. Specify whether all results that were compatible with each outcome domain in each study were sought (e.g. for all measures, time points, analyses), and if not, the methods used to decide which results to collect.	3-4
	10b	List and define all other variables for which data were sought (e.g. participant and intervention characteristics, funding sources). Describe any assumptions made about any missing or unclear information.	3-4
Study risk of bias assessment	11	Specify the methods used to assess risk of bias in the included studies, including details of the tool(s) used, how many reviewers assessed each study and whether they worked independently, and if applicable, details of automation tools used in the process.	4-5 Supplemental Table S1 and S2
Effect measures	12	Specify for each outcome the effect measure(s) (e.g. risk ratio, mean difference) used in the synthesis or presentation of results.	4
Synthesis methods	13a	Describe the processes used to decide which studies were eligible for each synthesis (e.g. tabulating the study intervention characteristics and comparing against the planned groups for each synthesis (item #5)).	4-5, Figure 1, Table 2
	13b	Describe any methods required to prepare the data for presentation or synthesis, such as handling of missing summary statistics, or data conversions.	4-5
	13c	Describe any methods used to tabulate or visually display results of individual studies and syntheses.	4-5, Figure 2, 3 and 4
	13d	Describe any methods used to synthesize results and provide a rationale for the choice(s). If meta-analysis was performed, describe the model(s), method(s) to identify the presence and extent of statistical heterogeneity, and software package(s) used.	4-5
	13e	Describe any methods used to explore possible causes of heterogeneity among study results (e.g. subgroup analysis, meta-regression).	5

Section and Topic	Item	Checklist item	Location where item is reported (p)
	13f	Describe any sensitivity analyses conducted to assess robustness of the synthesized results.	5
Reporting bias assessment	14	Describe any methods used to assess risk of bias due to missing results in a synthesis (arising from reporting biases).	4-5，Supplemental Figure S7
Certainty assessment	15	Describe any methods used to assess certainty (or confidence) in the body of evidence for an outcome.	4, Supplemental Table S6
**RESULTS**			
Study selection	16a	Describe the results of the search and selection process, from the number of records identified in the search to the number of studies included in the review, ideally using a flow diagram.	5, Figure 1
	16b	Cite studies that might appear to meet the inclusion criteria, but which were excluded, and explain why they were excluded.	5
Study characteristics	17	Cite each included study and present its characteristics.	5,Table 1
Risk of bias in studies	18	Present assessments of risk of bias for each included study.	Supplemental Table S4 and 5, Figure S7
Results of individual studies	19	For all outcomes, present, for each study: (a) summary statistics for each group (where appropriate) and (b) an effect estimate and its precision (e.g. confidence/credible interval), ideally using structured tables or plots.	8-10, Figure 2, 3 and 4
Results of	20a	For each synthesis, briefly summarise the characteristics and risk of bias among contributing studies.	8-10, Figure

Section and Topic	Item	Checklist item	Location where item is reported (p)
syntheses			2, 3 and 4
	20b	Present results of all statistical syntheses conducted. If meta-analysis was done, present for each the summary estimate and its precision (e.g. confidence/credible interval) and measures of statistical heterogeneity. If comparing groups, describe the direction of the effect.	8-10, Figure 2, 3 and 4, Supplemental Figure S1-S6
	20c	Present results of all investigations of possible causes of heterogeneity among study results.	10, Table 2
	20d	Present results of all sensitivity analyses conducted to assess the robustness of the synthesized results.	10
Reporting biases	21	Present assessments of risk of bias due to missing results (arising from reporting biases) for each synthesis assessed.	10
Certainty of evidence	22	Present assessments of certainty (or confidence) in the body of evidence for each outcome assessed.	8-10, Supplemental Table S5
**DISCUSSION**			
Discussion	23a	Provide a general interpretation of the results in the context of other evidence.	11
	23b	Discuss any limitations of the evidence included in the review.	11-12
	23c	Discuss any limitations of the review processes used.	12-13
	23d	Discuss implications of the results for practice, policy, and future research.	13
**OTHER INFORMATION**			
Registration and	24a	Provide registration information for the review, including register name and registration number, or state that the review was not registered.	3

Section and Topic	Item	Checklist item	Location where item is reported (p)
protocol	24b	Indicate where the review protocol can be accessed, or state that a protocol was not prepared.	3
	24c	Describe and explain any amendments to information provided at registration or in the protocol.	3
Support	25	Describe sources of financial or non-financial support for the review, and the role of the funders or sponsors in the review.	13
Competing interests	26	Declare any competing interests of review authors.	13
Availability of data, code and other materials	27	Report which of the following are publicly available and where they can be found: template data collection forms; data extracted from included studies; data used for all analyses; analytic code; any other materials used in the review.	13

From: Page MJ, McKenzie JE, Bossuyt PM, Boutron I, Hoffmann TC, Mulrow CD, et al. The PRISMA 2020 statement: an updated guideline for reporting systematic reviews. BMJ 2021;372:n71. doi: 10.1136/bmj.n71.

This work is licensed under CC BY 4.0. To view a copy of this license, visit https://creativecommons.org/licenses/by/4.0/

Table S3. Search strategy

Data source	Search terms
PubMed<br>2025 Dec 12<br>N=110	#1 (eat lancet diet[Title/Abstract]) OR (eat lancet[Title/Abstract]) OR (planetary health diet[Title/Abstract]) OR (plant diet[Title/Abstract]) OR (eat lancet healthy reference diet[Title/Abstract]) OR (plant based diet[Title/Abstract]) OR (EAT-HRD[Title/Abstract]) OR (vegetarian Diet[Title/Abstract]) OR (EAT-LDP[Title/Abstract]) OR (eat lancet reference diet[Title/Abstract]) OR (eat lancet diet pattern[Title/Abstract])<br>#2 (Depression [Title/Abstract]) OR (Depressive Disorder[Title/Abstract])<br>#3 (Anxiety[Title/Abstract]) OR (Anxieties[Title/Abstract])<br>#4 #2 OR #3<br>#5 #1 AND #4
Web of science<br>2025 Dec 12<br>N=675	#1 'eat lancet diet' (All Fields) or 'eat lancet' (All Fields) or 'planetary health diet' (All Fields) or 'plant diet' (All Fields) or 'eat lancet healthy reference diet' (All Fields) or 'plant based diet' (All Fields) or 'EAT-HRD' (All Fields) or 'vegetarian Diet' (All Fields) or 'EAT-LDP' (All Fields) or 'eat lancet reference diet' (All Fields) or 'eat lancet diet pattern' (All Fields)<br>#2 Depression (All Fields) or 'Depressive Disorder' (All Fields)<br>#3 Anxiety (All Fields) or Anxieties (All Fields)<br>#4 #2 OR #3<br>#5 #1 AND #4
EMBASE<br>2025 Dec 12<br>N=162	1 ('eat lancet diet' or 'eat lancet' or 'planetary health diet' or 'plant diet' or 'eat lancet healthy reference diet' or 'plant based diet' or 'EAT-HRD' or 'vegetarian Diet' or 'EAT-LDP' or 'eat lancet reference diet' or 'eat lancet diet pattern').ab,kw,ti.<br>2 (Depression or 'Depressive Disorder').ab,kw,ti.<br>3 (Anxiety or Anxieties).ab,kw,ti.<br>4 2 or 3<br>5 1 and 4
Cochrane Library<br>2025 Dec 12<br>N=134	#1 ('eat lancet diet'):ti,ab,kw OR ('eat lancet'):ti,ab,kw OR ('planetary health diet'):ti,ab,kw OR ('plant diet'):ti,ab,kw OR ('eat lancet healthy reference diet'):ti,ab,kw OR ('plant based die'):ti,ab,kw OR ('EAT-HRD'):ti,ab,kw OR ('vegetarian Diet'):ti,ab,kw OR ('EAT-LDP'):ti,ab,kw OR ('eat lancet reference diet'):ti,ab,kw OR ('eat lancet diet pattern'):ti,ab,kw<br>#2 (Depression):ti,ab,kw OR ('Depressive Disorder'):ti,ab,kw<br>#3 (Anxiety):ti,ab,kw OR (Anxieties):ti,ab,kw<br>#4 #2 or #3<br>#5 #1 and #4

Table S4. Quality assessment using the Newcastle-Ottawa scale for cross-sectional studies

Study ID	Selection Bias Assessment (Maximum 5 Stars)								Comparability (Maximum 2 Stars)		Outcome (Maximum 3 Stars)				Total Score (Maximum 10 Stars)
	Representativeness of the Sample		Sample Size		Non-Responders		Ascertainment of the Exposure (Risk Factor)		Confounding Factors are Controlled		Assessment of the Outcome		Statistical test		
	Selection	Score	Selection	Score	Selection	Score	Selection	Score	Selection	Score	Selection	Score	Selection	Score	
Tan et al. 2025	A	1	A	1	A	1	C	0	A	2	A	2	A	1	8
Tabatabaei et al.2025	A	1	A	1	A	1	C	0	A	2	A	2	A	1	8
Kamrani et al.2024	C	0	A	1	A	1	C	0	A	2	A	2	A	1	7
Jiang et al.2025	A	1	A	1	A	1	C	0	A	2	A	2	A	1	8
Bhering Martins et al. 2024	B	1	A	1	C	0	C	0	A	2	A	2	B	0	6

Table S5. Quality assessment using the Newcastle-Ottawa scale for cohort studies

Study ID	Selection Bias Assessment (Maximum 4 Stars)								Comparability (Maximum 1 Star)		Outcome (Maximum 3 Stars)						Total Score (Maximum 10 Stars)
	Representativeness of the Exposed Cohort		Selection of the Non-Exposed Cohort		Ascertainment of Exposure		Demonstration that outcome of Interest was not Present at Start of Study		Comparability of Cohorts on the Basis of the Design or Analysis		Assessment of the Outcome		Was Follow-Up Long Enough for Outcomes to Occur		Adequacy of Follow-Up of Cohorts		
	Selection	Score	Selection	Score	Selection	Score	Selection	Score	Selection	Score	Selection	Score	Selection	Score	Selection	Score	
Shi et al. 2025	A	1	A	1	B	1	A	1	A	1	B	1	A	1	D	0	7
Lu et al. 2024	A	1	A	1	B	1	A	1	A	1	B	1	A	1	D	0	7
Lan et al. 2025	A	1	A	1	B	1	A	1	A	1	B	1	A	1	D	0	7

Table S6. GRADEPro assessment of the certainty of evidence regarding ELD adherence and its association with depression and anxiety

Certainty Assessment							Effect		Certainty
No. of Studies	Study Design	Risk of Bias	Inconsistency	Indirectness	Imprecision	Other Considerations	Relative (95% CI)	Absolute (95% CI)	
ELD adherence and depression									
8	Nonrandomized studies	Not serious	Serious[a]	Not serious	Not serious	The funnel plot revealed pronounced asymmetry, suggesting the potential for publication bias[b]. Large-scale studies observed a significant dose-response gradient through trend tests or restricted cubic spline (RCS) curves[c].		OR=0.78 (0.73 to 0.85)	⊕⊕⊕◯ Moderate[abc]
ELD adherence and anxiety									
4	Nonrandomized studies	Not serious	Not serious	Not serious	Not serious	Large-scale study observed a significant dose-response gradient through trend tests or restricted cubic spline (RCS) curves[d].		OR=0.78 (0.57 to 1.05)	⊕⊕⊕◯ Moderate[d]
ELD adherence co-occurrence of depression and anxiety									
1	Nonrandomized studies	Not serious	Serious[e]	Not serious	Serious[f]	No effect was observed dose response gradient.		RR=0.76 (0.68 to 0.85)	⊕◯◯◯ Very low[f]

[a] :Downgraded 1 level for inconsistency, as substantial heterogeneity among the included studies(I^2=85.0%).

[b] :Downgraded 1 level for publication bias, as the funnel plot revealed pronounced asymmetry, suggesting the potential for publication bias.

[c]: Upgraded 1 level for dose-response gradient, as significant dose-response gradients were observed through trend tests or RCS curves in large-scale studies.

[d] :Upgraded 1 level for dose-response gradient, as significant dose-response gradients were observed through trend tests or RCS curves in large-scale study.

[e] :Not downgraded for inconsistency, as consistency could not be evaluated with only one study included.

[f] :Downgraded 1 level for imprecision, as only one study was included; despite an adequate sample size, the stability and reproducibility of the results remain uncertain.

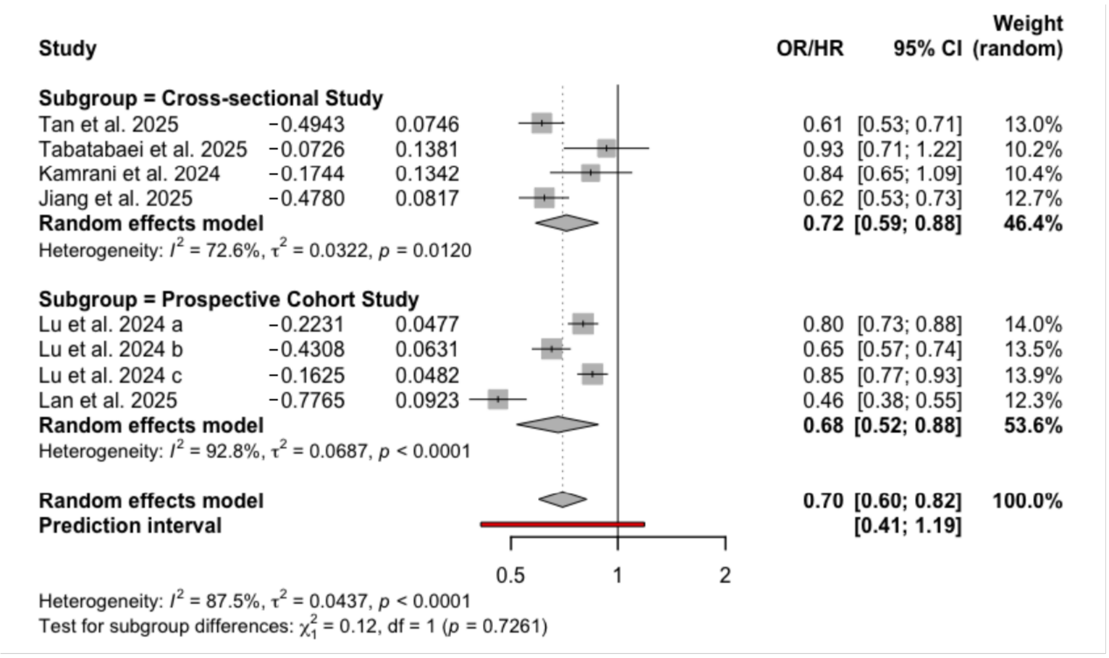

Figure S1. Forest plot of unadjusted associations of ELD and depression

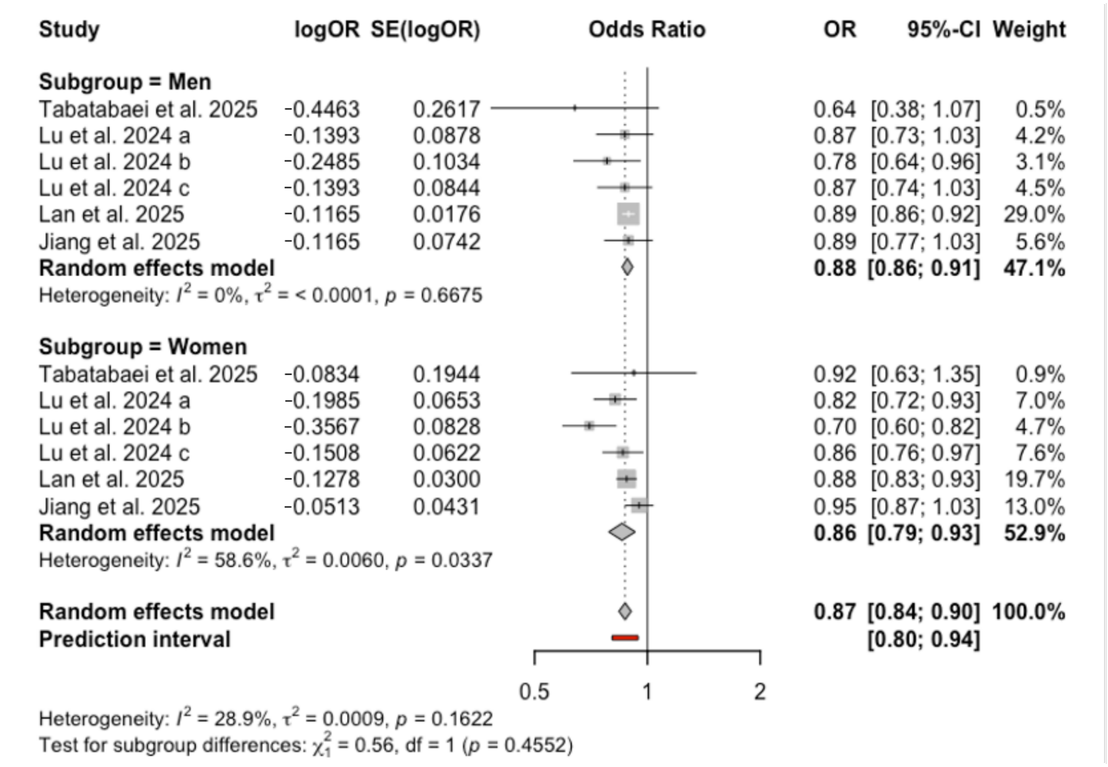

Figure S2. Forest plot of adjusted associations of ELD and depression by gender

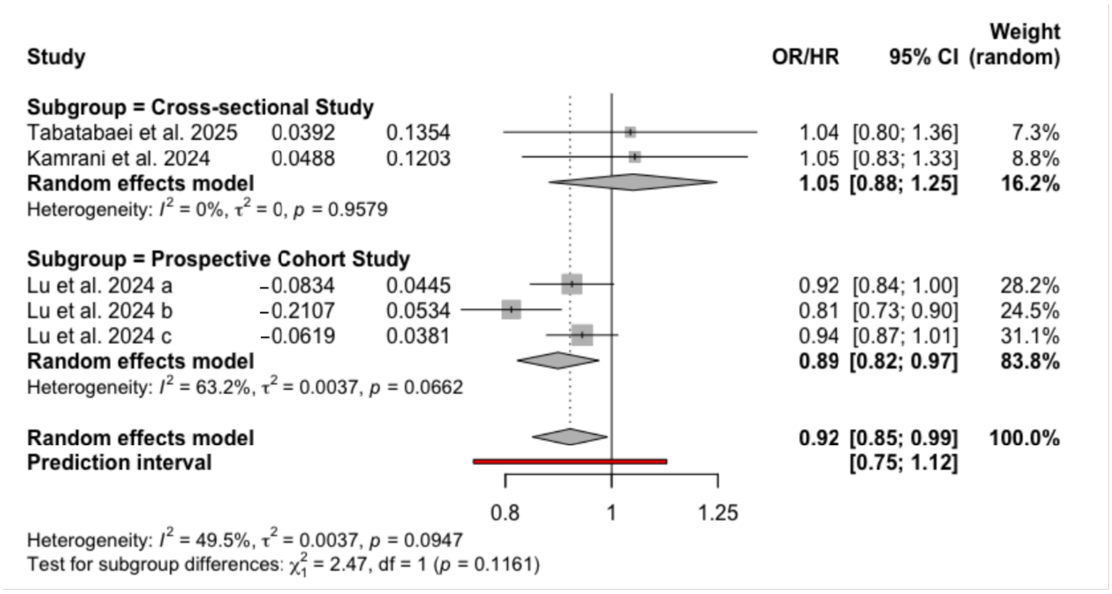

Figure S3. Forest plot of unadjusted associations of ELD and anxiety

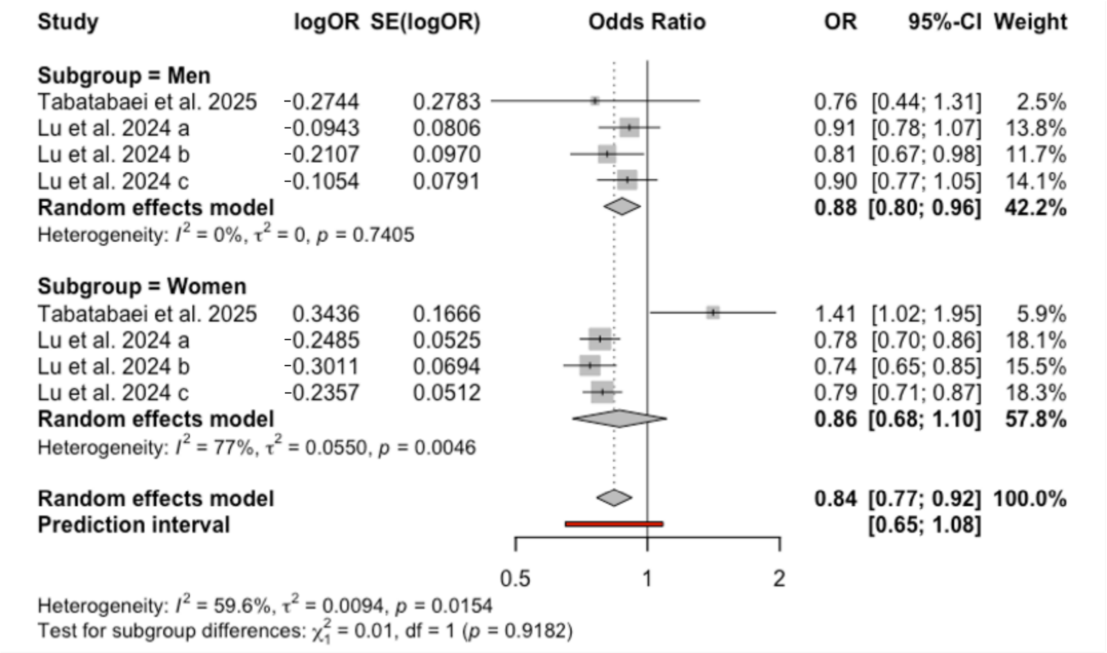

Figure S4. Forest plot of adjusted associations of ELD and anxiety by gender

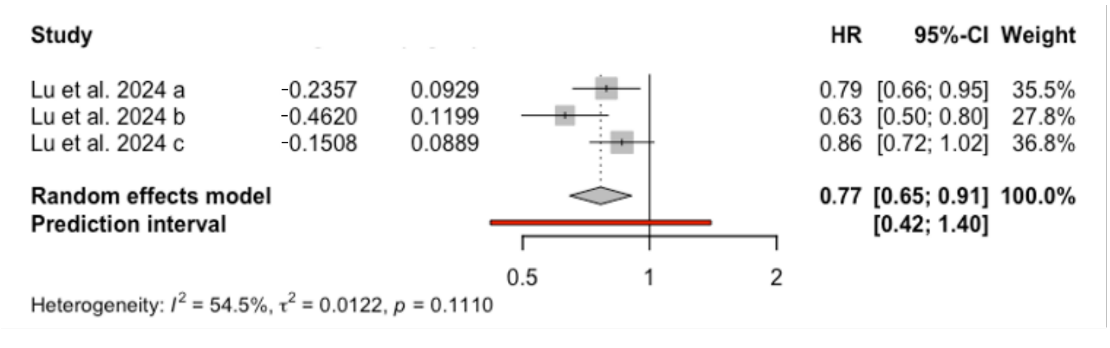

Figure S5. Forest plot of unadjusted associations of ELD and co-occurrence

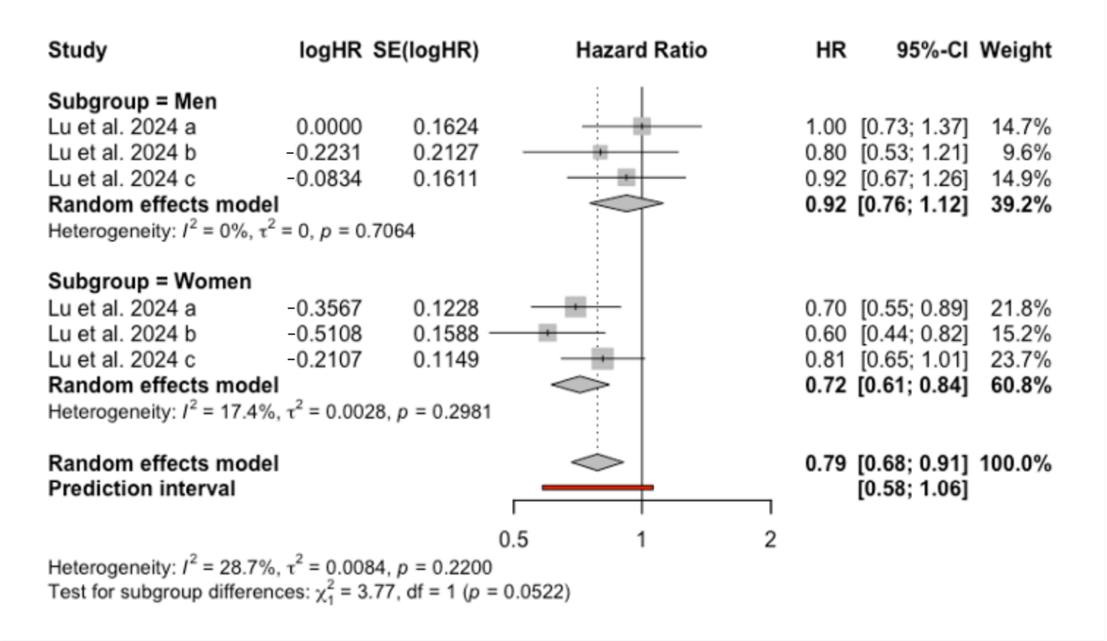

Figure S6. Forest plot of adjusted associations of ELD and co-occurrence by gender

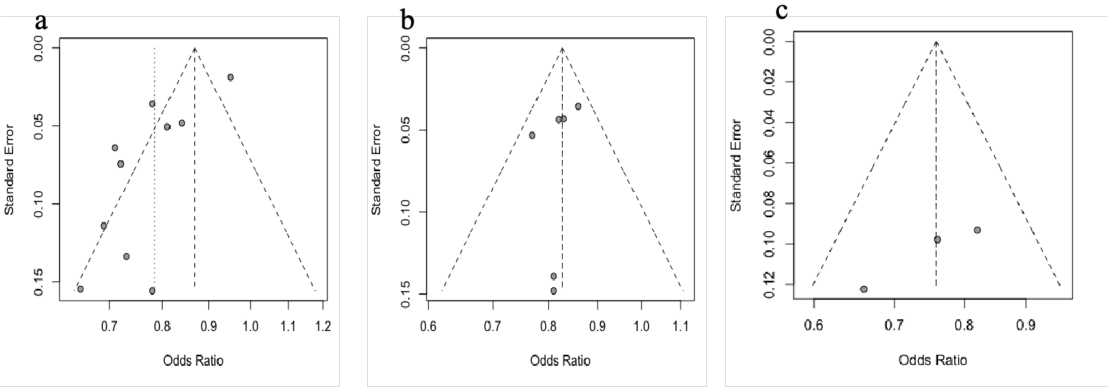

a. depression; b. anxiety; c. co-occurrence

Figure S7. Funnel plots for assessing publication bias in studies on ELD and depression/anxiety and co-occurrence
